# exoRBase 2.0: an atlas of mRNA, lncRNA and circRNA in extracellular vesicles from human biofluids

**DOI:** 10.1093/nar/gkab1085

**Published:** 2021-11-19

**Authors:** Hongyan Lai, Yuchen Li, Hena Zhang, Jia Hu, Jiatao Liao, Ying Su, Qin Li, Bing Chen, Caiping Li, Zhen Wang, Yan Li, Jialei Wang, Zhiqiang Meng, Zhaohui Huang, Shenglin Huang

**Affiliations:** Department of Integrative Oncology, Fudan University Shanghai Cancer Center, and Shanghai Key Laboratory of Medical Epigenetics, International Co-laboratory of Medical Epigenetics and Metabolism (Ministry of Science and Technology), Institutes of Biomedical Sciences, Fudan University, Shanghai, China; Department of Oncology, Shanghai Medical College, Fudan University, Shanghai, China; Department of Integrative Oncology, Fudan University Shanghai Cancer Center, and Shanghai Key Laboratory of Medical Epigenetics, International Co-laboratory of Medical Epigenetics and Metabolism (Ministry of Science and Technology), Institutes of Biomedical Sciences, Fudan University, Shanghai, China; Department of Oncology, Shanghai Medical College, Fudan University, Shanghai, China; Department of Integrative Oncology, Fudan University Shanghai Cancer Center, and Shanghai Key Laboratory of Medical Epigenetics, International Co-laboratory of Medical Epigenetics and Metabolism (Ministry of Science and Technology), Institutes of Biomedical Sciences, Fudan University, Shanghai, China; Department of Oncology, Shanghai Medical College, Fudan University, Shanghai, China; Department of Integrative Oncology, Fudan University Shanghai Cancer Center, and Shanghai Key Laboratory of Medical Epigenetics, International Co-laboratory of Medical Epigenetics and Metabolism (Ministry of Science and Technology), Institutes of Biomedical Sciences, Fudan University, Shanghai, China; Department of Oncology, Shanghai Medical College, Fudan University, Shanghai, China; Department of Medical Oncology, Fudan University Shanghai Cancer Center, Shanghai, China; Department of Oncology, Shanghai Medical College, Fudan University, Shanghai, China; Department of Integrative Oncology, Fudan University Shanghai Cancer Center, and Shanghai Key Laboratory of Medical Epigenetics, International Co-laboratory of Medical Epigenetics and Metabolism (Ministry of Science and Technology), Institutes of Biomedical Sciences, Fudan University, Shanghai, China; Department of Oncology, Shanghai Medical College, Fudan University, Shanghai, China; Department of Integrative Oncology, Fudan University Shanghai Cancer Center, and Shanghai Key Laboratory of Medical Epigenetics, International Co-laboratory of Medical Epigenetics and Metabolism (Ministry of Science and Technology), Institutes of Biomedical Sciences, Fudan University, Shanghai, China; Department of Oncology, Shanghai Medical College, Fudan University, Shanghai, China; Department of Integrative Oncology, Fudan University Shanghai Cancer Center, and Shanghai Key Laboratory of Medical Epigenetics, International Co-laboratory of Medical Epigenetics and Metabolism (Ministry of Science and Technology), Institutes of Biomedical Sciences, Fudan University, Shanghai, China; Department of Oncology, Shanghai Medical College, Fudan University, Shanghai, China; Department of Gastroenterology, Second Affiliated Hospital of Fujian Medical University, Quanzhou, China; Department of Integrative Oncology, Fudan University Shanghai Cancer Center, and Shanghai Key Laboratory of Medical Epigenetics, International Co-laboratory of Medical Epigenetics and Metabolism (Ministry of Science and Technology), Institutes of Biomedical Sciences, Fudan University, Shanghai, China; Department of Oncology, Shanghai Medical College, Fudan University, Shanghai, China; Department of Integrative Oncology, Fudan University Shanghai Cancer Center, and Shanghai Key Laboratory of Medical Epigenetics, International Co-laboratory of Medical Epigenetics and Metabolism (Ministry of Science and Technology), Institutes of Biomedical Sciences, Fudan University, Shanghai, China; Department of Oncology, Shanghai Medical College, Fudan University, Shanghai, China; Department of Medical Oncology, Fudan University Shanghai Cancer Center, Shanghai, China; Department of Oncology, Shanghai Medical College, Fudan University, Shanghai, China; Department of Integrative Oncology, Fudan University Shanghai Cancer Center, and Shanghai Key Laboratory of Medical Epigenetics, International Co-laboratory of Medical Epigenetics and Metabolism (Ministry of Science and Technology), Institutes of Biomedical Sciences, Fudan University, Shanghai, China; Department of Oncology, Shanghai Medical College, Fudan University, Shanghai, China; Wuxi Cancer Institute, Affiliated Hospital of Jiangnan University, Wuxi, China; Department of Integrative Oncology, Fudan University Shanghai Cancer Center, and Shanghai Key Laboratory of Medical Epigenetics, International Co-laboratory of Medical Epigenetics and Metabolism (Ministry of Science and Technology), Institutes of Biomedical Sciences, Fudan University, Shanghai, China; Department of Oncology, Shanghai Medical College, Fudan University, Shanghai, China

## Abstract

Extracellular vesicles (EVs) are small membranous vesicles that contain an abundant cargo of different RNA species with specialized functions and clinical implications. Here, we introduce an updated online database (http://www.exoRBase.org), exoRBase 2.0, which is a repository of EV long RNAs (termed exLRs) derived from RNA-seq data analyses of diverse human body fluids. In exoRBase 2.0, the number of exLRs has increased to 19 643 messenger RNAs (mRNAs), 15 645 long non-coding RNAs (lncRNAs) and 79 084 circular RNAs (circRNAs) obtained from ∼1000 human blood, urine, cerebrospinal fluid (CSF) and bile samples. Importantly, exoRBase 2.0 not only integrates and compares exLR expression profiles but also visualizes the pathway-level functional changes and the heterogeneity of origins of circulating EVs in the context of different physiological and pathological conditions. Our database provides an attractive platform for the identification of novel exLR signatures from human biofluids that will aid in the discovery of new circulating biomarkers to improve disease diagnosis and therapy.

## INTRODUCTION

Extracellular vesicles (EVs) are nano- to micrometer-sized lipid membrane vesicles (mainly including exosomes and microvesicles) secreted by virtually all cell types (1,2). These vesicles are highly abundant in blood-derived plasma/serum, urine, cerebrospinal fluid (CSF), bile, saliva and other human biofluids (3,4). EVs are widely involved in various physiological and pathological processes through the delivery of different types of bioactive molecules, including nucleic acids (especially RNAs), proteins, lipids and metabolites (5–7). Researchers have devoted substantial efforts to elucidate the biological properties of EV small RNAs (especially microRNAs) and their roles in diseases. Notably, emerging studies have shown that EVs in the circulatory system also contain different long RNA species, including messenger RNA (mRNA), long non-coding RNA (lncRNA) and circular RNA (circRNA) (3,8,9). These EV long RNAs (termed exLRs) are protected from degradation by the bilayer lipid membrane structure and exist stably in human biofluids. ExLRs approximately reflect the intracellular status of their host cells, which implies their potential roles as noninvasive biomarkers for the early detection and therapeutic evaluation of many complex disorders, especially cancers (8,10–12).

The application of advanced high-throughput RNA sequencing (RNA-seq) techniques has enabled researchers to comprehensively characterize the whole transcriptomic profiles of exLRs in large samples. We have developed an optimal strategy (termed exLR-seq) to extract and sequence exLRs, revealed abundant exLRs in human plasma and identified diverse specific markers potentially useful for cancer diagnosis (13,14). By combining exLR expression profiles with a robust algorithm, an EV deconvolution strategy (named EV-origin) was proposed to separately predict the relative and absolute fractions of EVs from blood cells and tissues (15). This traceability system makes it possible to decipher the complex heterogeneity of the tissue-cellular origin of circulating EVs. Furthermore, recent reports of EV transcriptomic characterization have also enabled the identification of specific exLRs for glioblastoma multiforme and defined different exLR panels for diagnosing esophageal squamous cell carcinoma and prostate cancer (16–18). Through differential gene expression and pathway enrichment analyses based on exLR profiles, Shi *et al.* monitored tumor-intrinsic and host immune status and predicted melanoma checkpoint blockade outcome (19). Progress in the study of EV transcriptome has emphasized the need for integrating and comparing the exLR profiles from healthy samples and different cancerous samples.

In 2017, we established exoRBase, a repository of exLRs in human blood exosomes from 92 normal controls and patients with five different cancer types (20). ExoRBase provides users an easy-to-use resource to query the annotation and expression information of exLRs. This database has received >70 000 universal visitors and has been highly cited by researchers. To date, several other databases depositing EV RNAs have also been published. However, these databases only focused on well-studied small RNAs or a small part of exLRs. For example, EVAtlas houses the expression profiles of seven ncRNA types in EV samples from 24 human tissues/diseases (21). LncExpDB documents 1,538 exosomal lncRNAs differentially expressed across diverse biological conditions (22). ExoceRNA was built based on our exoRBase and serves as a repository of competing endogenous RNAs in blood exosomes, which only represent a subset of exoRBase lncRNAs and mRNAs (23).

Here, we introduce a completely expanded version, exoRBase 2.0, which contains 905 exLR-seq data of EVs from four types of human biofluids, including blood, urine, CSF and bile. These biofluid samples were collected from healthy individuals as well as patients with 13 types of cancer or other disease. All exLR-seq data were analyzed using an improved bioinformatics pipeline. The annotation information and expression profiles of 19 643 mRNAs, 15 645 lncRNAs and 79 084 circRNAs in EVs were obtained. We also covered the enrichment scores of 11 536 MSigDB (Molecular Signatures Database) pathways for each sample generated by ssGSEA (single sample Gene set Enrichment Analysis) analyses on exLR expression profiles. In addition, exoRBase 2.0 provides the relative and absolute distribution of 16 types of tissue cells and 23 types of blood cells produced by the modified EV-origin approach. All data and plots in exoRBase 2.0 are freely available for download.

## MATERIALS AND METHODS

### Integration of available RNA-seq data

We collected a total of 905 RNA-seq data of EVs from human blood, urine, CSF and bile samples, which were compiled into the exoRBase 2.0 database (Table 1). These blood samples were associated with diverse biological conditions, including healthy state, benign disease, breast cancer (BRCA), coronary heart disease (CHD), colorectal cancer (CRC), esophageal squamous cell carcinoma (ESCC), glioblastoma multiforme (GBM), gastric cancer (GC), kidney cancer (KIRC), hepatocellular carcinoma (HCC), malignant lymphoma (ML), melanoma (MEL), ovarian cancer (OV), pancreatic adenocarcinoma (PAAD) and small cell lung cancer (SCLC). The source of each dataset was listed in the website (http://www.exorbase.org/exoRBaseV2/statistics/index). The latest release (RNA-seq analysis V8) of gene expression TPM profiles (GENCODE version 26) across 30 tissues in the Genotype-Tissue Expression (GTEx) project was also downloaded for annotating possible original tissues of exLRs (24).

**Table 1.** Expanded data in exoRBase 2.0 compared with exoRBase 1.0

Type	Cohort	exoRBase 1.0	exoRBase 2.0
Urine	Urine	_	16
CSF	CSF	_	5
Bile	Bile	_	17
Blood	Healthy	32	118
	Benign	_	130
	BRCA	2	140
	CHD	6	12
	CRC	12	35
	ESCC	_	6
	GBM	_	13
	GC	_	9
	HCC	21	112
	KIRC	_	15
	ML	_	28
	MEL	_	21
	OV	_	30
	PAAD	14	164
	SCLC	_	36
Target	mRNA	18 333	19 643
	lncRNA	15 501	15 645
	circRNA	58 330	79 084
	Pathway	_	11 536
	Tissue/Cell origin	_	39

CSF, cerebrospinal fluid; BRCA, breast cancer; CHD, coronary heart disease; CRC, colorectal cancer; ESCC, esophageal squamous cell carcinoma; GBM, glioblastoma multiforme; GC, gastric cancer; KIRC, kidney cancer; HCC, hepatocellular carcinoma; ML, malignant lymphoma; MEL, melanoma; OV, ovarian cancer; PAAD, pancreatic adenocarcinoma; SCLC, small cell lung cancer.

### Identification, annotation and quantification of exLRs with an improved pipeline

**Figure 1.**
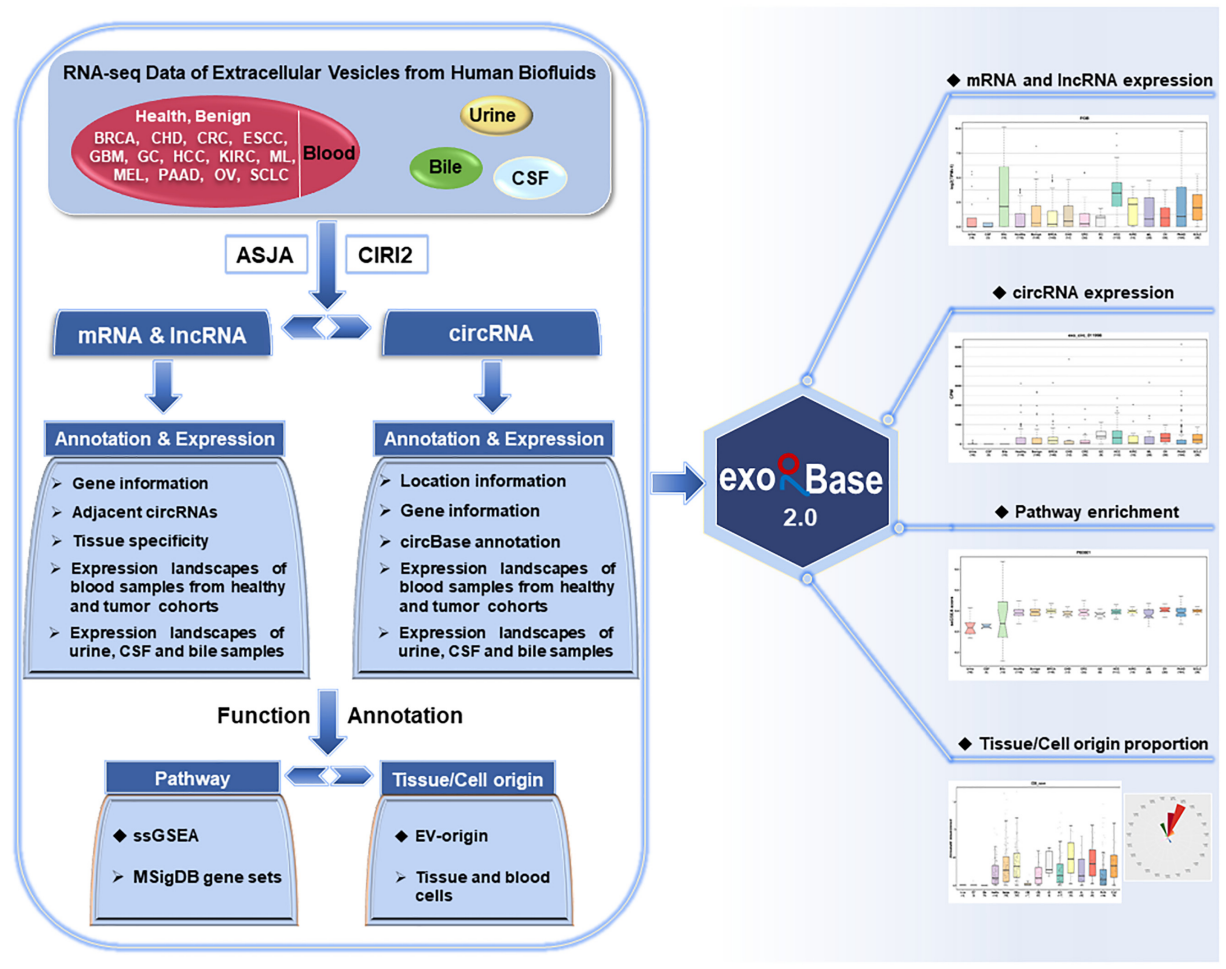
A schematic overview of the exoRBase 2.0 core content and framework. ExoRBase 2.0 integrates RNA-seq data of EVs from human blood, urine, CSF and bile samples. The exLRs, including mRNAs, lncRNAs and circRNAs, were identified, annotated and quantified according to the Assembling Splice Junctions Analysis (ASJA) and CircRNA Identifier (CIRI2) bioinformatic tools. To interpret EV mRNA expression profiles, pathway enrichment analysis was performed on MSigDB gene sets using the ssGSEA method. The EV-origin approach was applied to predict the proportions of tissue/blood cell sources. ExoRBase 2.0 visualizes and compares exLR expression profiles as well as the enrichment levels of functional pathways and the origins of circulating EVs.

Referring to the ASJA program (Assembling Splice Junctions Analysis, https://github.com/HuangLab-Fudan/ASJA) (25), we employed an improved exLR-seq analysis pipeline to reanalyze the raw sequencing data of all samples (Figure 1). Briefly, the overall quality of raw FASTA files was assessed by FastQC (version 0.11.8) followed by filtering out low-quality reads and removing adapter sequences with the help of Trimmomatic (version 0.36) (26). The remaining reads were aligned to the human reference genome (GRCh38 from GENCODE) by STAR (version 2.7.1a) in 2-pass mapping mode (27). The reads mapped to regions of protein coding or long non-coding (mRNAs or lncRNAs) genes were counted by featureCounts (version 1.6.3) with an appropriate setting of strand-specific parameter ‘-s’ (28). The read count of each gene was normalized to the TPM (transcripts per million) value as follows: $TP{M_i} = \frac{{R{C_i}{\rm{\;}}/{\rm{\;}}{L_i}{\rm{\;*\;}}{{10}^6}}}{{\mathop \sum \nolimits_{j = 1}^N R{C_j}{\rm{\;}}/{\rm{\;}}{L_j}}}\;$, where $R{C_i}$ is the count of reads mapped to gene $i$, and ${L_i}$ is the length of gene $i$. $N$ denotes the number of all mRNA and lncRNA genes. The circRNAs were identified, annotated and quantified by the ASJA program (25). Additionally, the CIRI2 (CircRNA Identifier), an improved circRNA detection tool, was also used to recognize circRNAs for filtering false positives (29). The final circRNA set was determined as the circRNAs detected by both ASJA and CIRI2 algorithms. To eliminate the impact of sequencing depth on circRNA data, we calculated the CPM (counts per million) value for each circRNA through the number of reads mapped to a circRNA multiplied by 10^6^ and divided by the total number of mapped reads of a given sample.

The basic gene information for all exLRs was annotated with GENCODE version 29. An exLR detected in at least three blood samples would be annotated as blood exLR, and an exLR detected in at least 1 urine (or CSF, bile) sample was annotated as urine (or CSF, bile) exLR. The minimal expression threshold of exLRs was 0.1 and circRNAs had at least two reads. Based on the GTEx expression atlas (V8 release), the tissue specificity scores of mRNA and lncRNA genes were calculated as the difference between the maximum possible entropy and the Shannon entropy of expression values for a gene in all tissues (30,31). The circBase annotation information of circRNAs was obtained as previously described in exoRBase 1.0.

### Pathway enrichment analysis

To investigate pathway-level differences among different cohorts, we downloaded the gene sets annotated with pathways from MSigDB (version 7.2, https://www.gsea-msigdb.org/gsea/msigdb/) (32), including seven major collections: 50 hallmark gene sets (33), 292 BIOCARTA (34), 186 KEGG (35) and 1554 REACTOME (36) gene sets of canonical pathways, 7579 BP (biological process) and 1696 MF (molecular function) gene sets derived from the Gene Ontology (GO) resource (37), and 189 oncogenic signature gene sets of cellular pathways. The ssGSEA method implemented in the R GSVA package was then used to analyze the enrichment scores of these 11 536 MSigDB pathways for each sample based on exLR expression profiles (38,39).

### Enumerating tissue-cellular origin of EVs

The estimation analysis of tissue-cellular source contributions of EVs for each sample was performed using EV-origin (15). This approach implemented the quantification of EVs by combining signature genes of tissues and blood cells with expression profiles of exLRs. The EV-origin R program was modified and applied to separately predict the relative and absolute fractions of EVs derived from 16 human tissues (including adipose tissue, bladder, brain, colon, esophagus, heart, kidney, liver, lung, muscle, nerve, pancreas, pituitary, skin, small intestine and stomach) and 23 types of blood cells (including red blood cells, platelets, CD8+ naive T cells, central memory CD8+ T cells, effector memory CD8+ T cells, terminal effector CD8+ T cells, mucosal-associated invariant T cells, follicular helper T cells, T regulatory cells, type 1 T-helper cells, type 17 T-helper cells, type 2 T-helper cells, CD4+ naive T cells, naive B cells, plasmablasts, natural killer cells, neutrophils, basophils, terminal effector CD4+ T cells, memory B cells, dendritic cells, monocytes and γδ T cells). Table 2 provides the detailed description of all 39 types of EV origins included in exoRBase 2.0.

**Table 2.** The detailed description of all traceable tissue/cellular types in exoRBase 2.0

Tissue/cell name	Full name	Main category	Sub category
RBC	Red blood cells	Blood	HSC (hematopoietic stem cell)
Platelet	Platelet	Blood	HSC (hematopoietic stem cell)
CD8_naive	CD8+ naive T cells	Blood	Adaptive immunity
CD8_CM	Central memory CD8+ T cells	Blood	Adaptive immunity
CD8_EM	Effector memory CD8+ T cells	Blood	Adaptive immunity
CD8_TE	Terminal effector CD8+ T cells	Blood	Adaptive immunity
MAIT	Mucosal-associated invariant T cells	Blood	Innate immunity
TFH	Follicular helper T cells	Blood	Adaptive immunity
Treg	T regulatory cells	Blood	Adaptive immunity
Th1	Type 1 T-helper cells	Blood	Adaptive immunity
Th17	Type 17 T-helper cells	Blood	Adaptive immunity
Th2	Type 2 T-helper cells	Blood	Adaptive immunity
CD4_naive	CD4+ naive T cells	Blood	Adaptive immunity
B_naive	Naive B cells	Blood	Adaptive immunity
Plasmablasts	Plasmablasts	Blood	Adaptive immunity
NK	Natural killer cells	Blood	Innate immunity
Neutrophils	Neutrophils	Blood	Innate immunity
Basophils	Basophils	Blood	Innate immunity
CD4_TE	Terminal effector CD4+ T cells	Blood	Adaptive immunity
B_Memory	Memory B cells	Blood	Adaptive immunity
DCs	Dendritic cells	Blood	Innate immunity
Monocytes	Monocytes	Blood	Innate immunity
T_gd	γδ T cells	Blood	Innate immunity
Adipose Tissue	Adipose Tissue	Tissue	Other tissue
Bladder	Bladder	Tissue	Solid organ
Brain	Brain	Tissue	Solid organ
Colon	Colon	Tissue	Solid organ
Esophagus	Esophagus	Tissue	Solid organ
Heart	Heart	Tissue	Solid organ
Kidney	Kidney	Tissue	Solid organ
Liver	Liver	Tissue	Solid organ
Lung	Lung	Tissue	Solid organ
Muscle	Muscle	Tissue	Other tissue
Nerve	Nerve	Tissue	Other tissue
Pancreas	Pancreas	Tissue	Solid organ
Pituitary	Pituitary	Tissue	Solid organ
Skin	Skin	Tissue	Solid organ
Small Intestine	Small Intestine	Tissue	Solid organ
Stomach	Stomach	Tissue	Solid organ

### Statistical analysis and visualization for homogeneity or heterogenicity of targets

To evaluate the expression patterns of exLRs in various biofluids among different biological conditions, we calculated the expression frequencies (and corresponding sample numbers) and mean expression values of exLRs in urine, CSF, and bile samples as well as healthy, benign and tumor blood samples. The mean expression values of exLRs in different tumor cohorts were also calculated and further visualized using line and heat map charts. The mean enrichment scores of each pathway and mean absolute proportions of each EV source in all cohorts were also calculated and visualized. To characterize the differential expression or enrichment targets, the Mann–Whitney U test was used to separately perform differential analysis between healthy individuals and each disease/tumor cohort. To avoid the impact of sample imbalance on differential analysis, we randomly sampled 35 healthy samples to compare with groups with relatively fewer samples (CHD, CRC, GC, KIRC, ML, OV and SCLC). ExLRs with |log_2_FC (fold change)| > 1 and *P*-value <0.05 were considered significantly differential targets, and the enrichment results from pathway analyses and EV-origin strategy with p-value < 0.05 were also included in this study. For groups with larger sample sizes (benign, BRCA, HCC, PAAD), the *q-*values (adjusted using the adjusted Benjamini–Hochberg method) of differential targets were <0.05. Box plots were employed to exhibit or compare the homogeneous or heterogeneous patterns of exLRs as well as information on pathways and EV origins across all cohorts. In addition, a cumulative percentage chart and rose polar diagram were used to indicate the relative fractions of 16 tissue or 23 blood cell types of EV origins in each exLR-seq sample. All statistical analyses and plots in exoRBase 2.0 were generated using R software (version 4.0.2) and ggplot2 (version 3.3.2) and ComplexHeatmap (version 2.4.3) packages.

### Database construction

The exoRBase 2.0 database was developed based on Akka 2.6.5 (web server) and MySQL (database server). All data were organized and managed by MySQL (version 5.7.31), an open-source relational database management system. The interface of the website was designed and implemented using the Twirl template engine (version 1.5.0). The query system of this database was configured and handled by Play Slick (version 4.0.2). ExoRBase 2.0 can be successfully accessed by different web browsers, including Internet Explorer, Google Chrome, Firefox and Safari.

## UPDATED DATABASE CONTENT AND USER INTERFACER

Overall, exoRBase 2.0 focuses on integrating and characterizing the transcriptome data of EVs from human body fluids. A system-level overview of the workflow and kernel data for exoRBase 2.0 is presented in Figure 1. By performing a large-scale integration and bioinformatic analysis of EV exLR-seq datasets, all expressed exLRs were identified and annotated. Expression profiles of exLRs as well as enrichment levels of MSigDB pathways and tissue-cellular origins of EVs are stored in exoRBase 2.0 and can be visualized with different plots.

### Expanded data and new features

This updated database contains high-quality exLR-seq data of EVs in blood, urine, CSF and bile samples from 905 individuals. Compared with the previous version, urine (16 samples), CSF (5 samples) and bile (17 samples) represent novel biofluid types. Moreover, exoRBase 2.0 houses many more blood samples obtained from fifteen groups, including healthy participants (118 samples), patients with benign disease (130 samples), BRCA (140 samples), CHD (12 samples), CRC (35 samples), ESCC (6 samples), GBM (13 samples), GC (9 samples), HCC (112 samples), KIRC (15 samples), ML (28 samples), MEL (21 samples), OV (30 samples), PAAD (164 samples) and SCLC (36 samples). Through RNA-seq analysis, the annotation information and expression profiles of 19 643 mRNAs, 15 645 lncRNAs and 79 084 circRNAs detected in EVs were obtained and compiled into exoRBase 2.0. In particular, this database has added the enrichment scores of 11 536 MSigDB pathways and the relative and absolute enrichment proportions of 39 tissue-cellular components of circulating EVs for each sample, which are generated based on the exLR expression profiles. Table 1 summarizes the increased data and novel contents in exoRBase 2.0.

### The enhanced browse, search and detail platform

**Figure 2.**
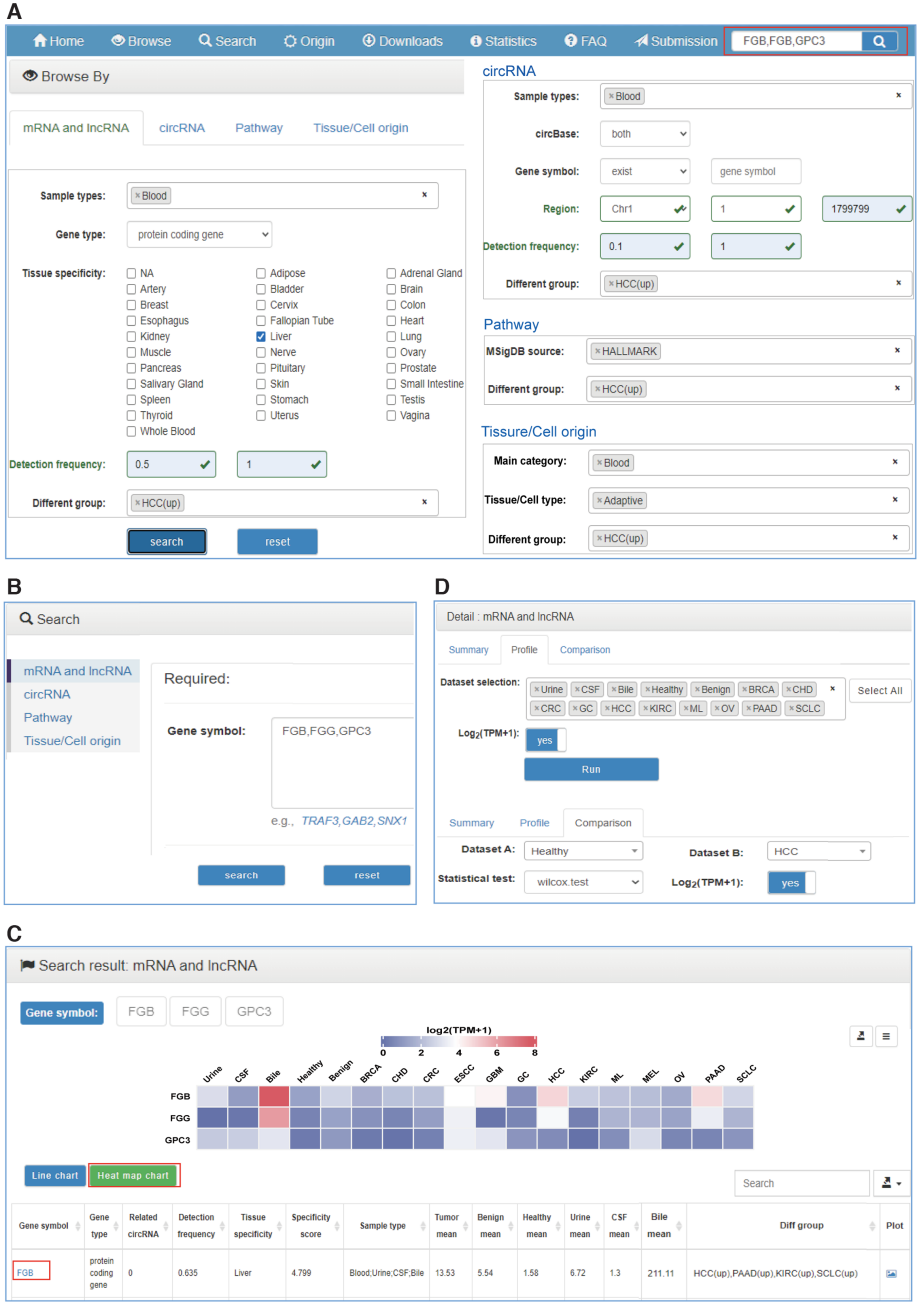
The enhanced user interface of exoRBase 2.0. (**A**) The browse pages for mRNA, lncRNA, circRNA, pathway and tissue/cell origin. (**B**) The search section with four search pages. (**C**) The search result page with line and heat map charts and a result table. ‘Tumor mean’, ‘Benign mean’ and ‘Healthy mean’ represent the average expression/enrichment values of blood samples from tumor, benign and healthy cohorts, respectively. ‘Urine mean’, ‘CSF mean’ and ‘Bile mean’ indicate average values for urine, CSF and bile samples, respectively. The ‘Different group’ shows significantly divergent groups compared with healthy individuals. (**D**) The detail section of each target containing ‘Summary’, ‘Profile’ and ‘Comparison’ pages.

The web interfaces of exoRBase 2.0 have been redesigned to make it more accessible and user-friendly. The major user interfaces are shown in Figure 2.

#### Browse pages

In the browse section, there are four web pages for users to browse mRNA, lncRNA, circRNA, Pathway or Tissue/Cell origin (Figure 2A). The browse pages for mRNA, lncRNA and circRNA display exLRs recognized in healthy or cancerous biofluids with their basic gene annotation information, as well as general expression information. Users can browse exLRs in this database with different filter criteria. For example, ‘Sample types’ enables users to browse exLRs expressed in single or multiple human biofluids. Users can select frequently expressed exLRs in all samples using the ‘Detection frequency’ input box or select differentially expressed exLRs in certain cancer types compared with healthy individuals using the ‘Different group’ drop-down box. Furthermore, ‘Gene type’ and ‘Tissue specificity’ offer the selection of tissue-specific expressed mRNAs and/or lncRNAs. Users can click the gene symbol to access to the detailed information of a mRNA or lncRNA and click the related circRNA hyperlink to obtain the circRNAs annotated to the corresponding mRNA or lncRNA gene. On the circRNA page, the novel or annotated circRNAs detected in specific genome regions can be screened based on ‘Gene symbol’ and ‘Region’ options. Clicking the circID and circBase ID displays detailed information on the circRNA contained in exoRBase 2.0 and circBase.

In the newly added two browse pages for Pathway and Tissue/Cell origin, the basic annotation and statistical information of pathways and origins are listed. The ‘Different group’ can be specified to choose significantly differential pathways or EV origins. We also provide the selective browsing of pathways that are recorded as specific ‘MSigDB source’. The 39 EV origins can also be selectively browsed according to the ‘Main category’ or ‘Tissue/Cell type’ annotation information. Clicking the pathway ID or tissue/cell name reveals the detail page for Pathway or Tissue/Cell origin. Users can click the pathway name to view the detailed information in the MSigDB database. We also offer a plot icon for every target to directly connect to the profile graph.

In the browse section, entries can be sorted by each column of the table in ascending or descending order. Users can manually set the number of entries displayed per page (default 10) and directly locate entries of interest by a fast, easy search with key words. The whole or filtered browse tables can be downloaded freely.

#### Search and results

By clicking the ‘Search’ tab on the top navigation menu, users will jump to the search section from any pages (Figure 2B). Users can search their mRNAs, lncRNAs, circRNAs, pathways or tissue/cell origins of interest by entering a comma-separated list of gene symbols, circRNA IDs, pathway IDs/names, or tissue/cell names on the corresponding search page. A simple search box on the right side of the top navigator on every page can be used to query mRNA or lncRNA genes quickly (Figure 2A). For circRNAs, users can also search for circRNAs annotated to specific genes by inputting gene symbols. The ‘Advanced filter’ denotes querying the circRNAs located in specific regions of one chromosome. After clicking the ‘Search’ button, a search result page that includes two charts and a table will be displayed (Figure 2C). The columns of the search result table are the same as those in the browsing table. The heat map chart and line chart demonstrate the mean TPM values (or CPM values, ssGSEA scores, absolute proportions) of each mRNA/lncRNA gene (or circRNA, Pathway, Tissue/Cell origin) across urine, CSF, bile and 15 groups of blood samples. Both the charts and data can be downloaded with different formats.

#### Detail pages

Clicking the gene symbol, circID, pathway ID or tissue/cell name in both browse and search result tables will link to the corresponding detail section that currently includes ‘Summary’, ‘Profile’ and ‘Comparison’ three pages (Figure 2D). For exLRs, users can view more comprehensive and detailed annotation information as well as the expression frequencies (sample numbers) and mean expression values in tumor, benign, healthy, urine, CSF and bile samples from the ‘Summary’ page. On the ‘Profile’ page, the expression profile of an exLR or the enrichment profile of a pathway across all biofluid sample groups will be visualized with a box plot. For an EV origin, both the absolute and relative enrichment proportion profiles are plotted. Users can manually select fewer groups of interest to be displayed. For exLRs, we provide the choice of log_2_ normalization with ‘Log_2_(TPM + 1)’ and ‘Log_2_(CPM + 1)’ boxes. On the ‘Comparison’ page, users can explore the difference between two groups with the Student's *t*-test (for groups with few samples) or Wilcoxon test (for groups with many samples). Similarly, the plots and data are also available for download.

### Extended contents and database utility

#### Application of mining exLR markers

**Figure 3.**
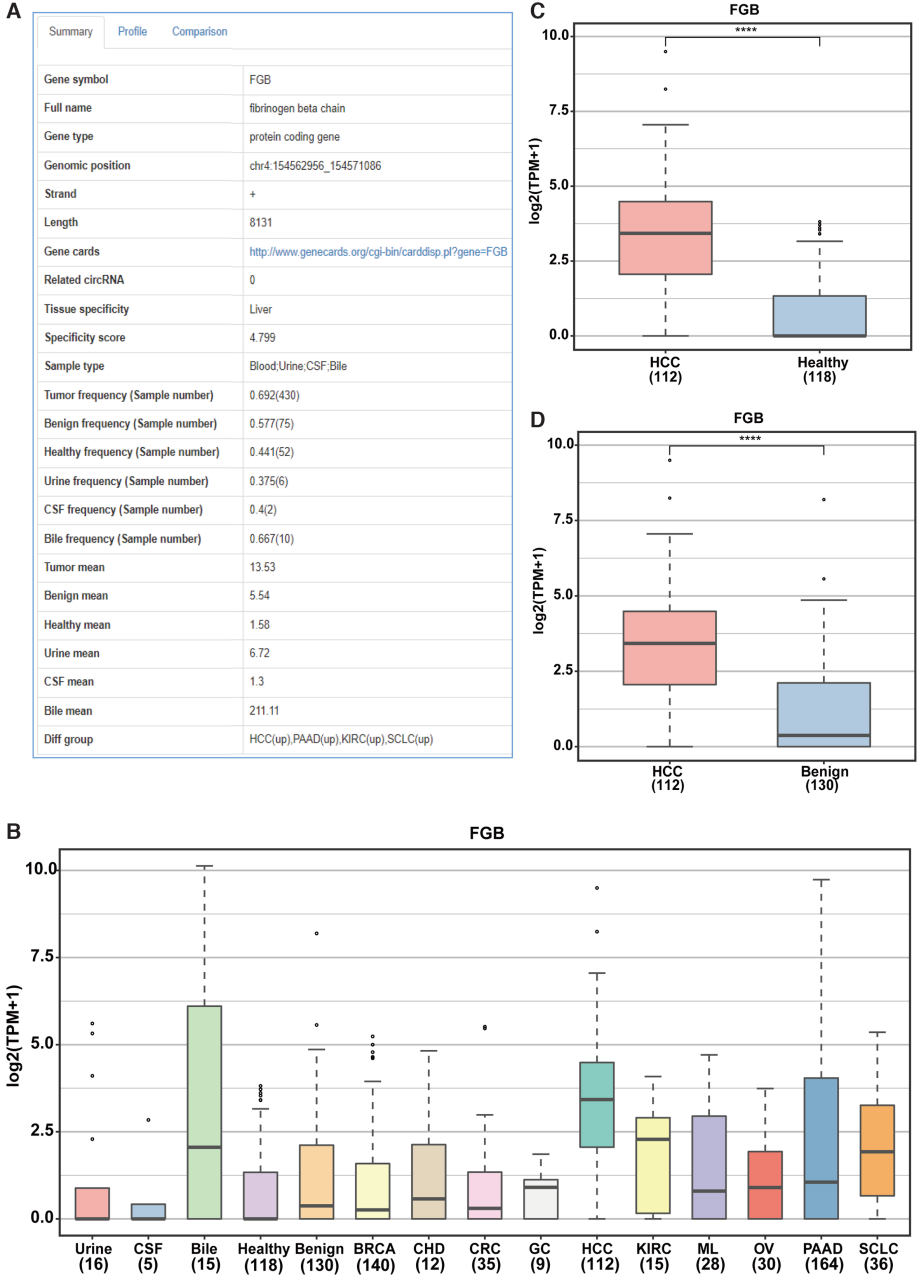
Detailed information and expression profile of FGB. (**A**) The ‘Summary’ information of FGB gene in the ‘Detail’ section. (**B**) The expression landscape of FGB across different groups incorporated into exoRBase 2.0 on the ‘Profile’ page. (**C, D**) Differential expression analysis of FGB between HCC and healthy/benign cohorts on the ‘Comparison’ page. Abbreviations: CSF, cerebrospinal fluid; BRCA, breast cancer; CHD, coronary heart disease; CRC, colorectal cancer; GC, gastric cancer; KIRC, kidney cancer; HCC, hepatocellular carcinoma; ML, malignant lymphoma; OV, ovarian cancer; PAAD, pancreatic adenocarcinoma; SCLC, small cell lung cancer.

Users can browse and search all exLRs in exoRBase 2.0 and can also mine candidate exLR biomarkers of specific biofluid types or cancer types. For example, the frequently overexpressed liver-specific mRNA genes in HCC blood samples can be screened out with the specified options (Sample types with ‘Blood’, Gene type with ‘protein coding gene’, Tissue specificity with ‘Liver’, Detection frequency with ‘0.5–1’, Different group with ‘HCC(up)’) on the browse page (Figure 2A). The greatly narrowed exLR set might exhibit potential as biomarkers for discriminating HCC patients from healthy individuals and can be evaluated and analyzed subsequently in other ways. More detailed information on each exLR can be obtained by clicking each gene symbol. In addition, users can directly query desired single or multiple exLRs on the search page. For example, the FGB, FGG and GPC3 genes have been reported as HCC EV-derived mRNA markers for noninvasive early detection of HCC (40). As a demonstration, we entered ‘FGB, FGG, GPC3’ into the box on the search page of mRNA and lncRNA or the simple search box on any page (Figure 2A, B). The search results include two charts and one table. The heat map chart provides a holistic view of the average expression level of the three exLRs across all cohorts (Figure 2C). By clicking the gene symbol ‘FGB’, detailed information on FGB can be obtained. From the ‘Summary’ page, we further find that FGB is frequently expressed in tumor, benign and healthy cohorts and is detected in most bile samples but in only a few urine and CSF samples (Figure 3A). The expression profile of FGB in urine, CSF, bile and 12 groups of blood samples is presented in Figure 3B. Moreover, the comparison between HCC and healthy as well as benign samples was performed using the Wilcox test in the ‘Comparison’ page. The results show that FGB is significantly upregulated in HCC patients (Figure 3C, D), which is consistent with previously reported results (40).

#### Applications to explore pathways and EV origins

Based on the exLR expression profiles, we further performed MSigDB pathway enrichment analysis and estimated the proportions of potential EV sources using EV-origin approach. These are two important modules of exoRBase 2.0. This improved database now enables users to study the homogeneity and heterogenicity of pathways and EV origins across different groups of biofluid samples. Users can explore desired pathways or EV origins on the browse and search pages in a manner similar to that described for exLRs.

**Figure 4.**
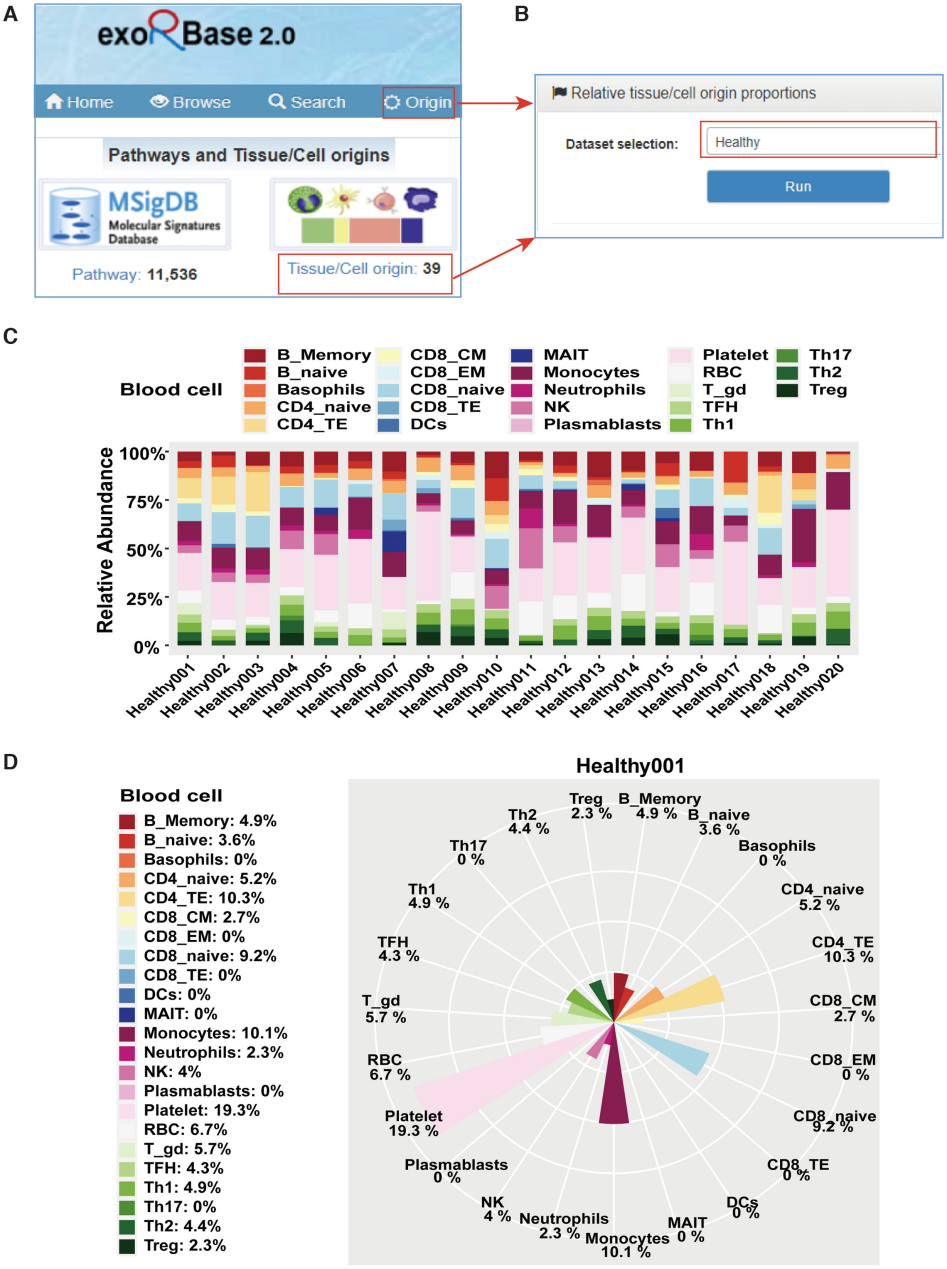
The relative proportions of EV origins. (**A**) The ‘Origin’ tab at the top of each page and the ‘Tissue/Cell origin’ tab on the home page for accessing to the relative information obtained from EV-origin method. (**B**) The ‘Relative tissue/cell origin proportions’ page with selection of the ‘Healthy’ dataset. (**C**) The relative abundances of 23 types of blood cells in the top 20 healthy samples. (**D**) The relative abundances of 23 types of blood cells in the Healthy001 sample. In the two charts, the fractions of blood cells in each sample are indicated by different colors, and the lengths of bars indicate the enrichment levels of blood cell populations.

ExoRBase 2.0 supports the intuitive visualization not only of the absolute proportions of tissue/cell origins for comparison between different cohorts but also of the relative proportions of tissue/cell origins for comparison between different tissue/cell origins in each sample. By clicking the ‘Origin’ on the navigation bar or the ‘Tissue/Cell origin’ on the home page, the ‘Relative tissue/cell origin proportions’ page is displayed (Figure 4A and B). By selecting a dataset of interest (e.g., Healthy) and clicking the ‘Run’ icon, the relative abundances of 16 tissue origins and 23 blood cell origins for the top 20 healthy samples are separately shown in two cumulative percentage charts. Taking the chart of blood cell origins as an example (Figure 4C), the relative proportions of EVs originating from the 23 blood cells in each healthy sample are presented in different colors, and the lengths of bars represent the levels of blood cell origins. In the chart, we observe that most heathy samples have a high percentage of EVs released by platelets. In addition, all healthy samples are listed in a table under the charts on this page. Clicking the ‘Tissue cells’ icon or ‘Blood cells’ icon of a given sample (e.g. Healthy001), a hover page will appear to display the relative proportions of this sample with a rose polar chart. Figure 4D suggests that higher levels of EVs are released by CD4_TE, CD8_naive, monocytes and platelets in the Healthy001 sample.

## DISCUSSION AND PERSPECTIVES

In this work, we introduce the greatly improved exoRBase 2.0, which documents a large set of exLRs expressed in 905 urine, CSF, bile and blood samples from healthy individuals and patients with benign diseases and tumors. This database enables users to query and visualize the comprehensive annotation information and expression landscapes of exLRs, the enrichment scores of MSigDB pathways and the relative and absolute fractions of tissue and blood cell origins of EVs. ExoRBase 2.0 serves not only as a resource for exLRs but also as a platform for assessing the potential functional changes of exLRs and the heterogeneity of tissue-cellular origins of EVs.

According to the professional practice recommended by The International Society for Extracellular Vesicles (ISEV), nanometer-sized membrane-bound particles (mainly including exosomes and microvesicles) are uniformly named extracellular vesicles (EVs). We followed this nomenclative principle by using the term EVs instead of exosomes in the updated database. ExoRBase 2.0 features several advantages compared with exoRBase 1.0. First, the new version provides a holistic view of EV transcriptomes in diverse human biofluids. Single exLR expression analysis can be performed among more than ten disease/tumor cohorts. Second, exoRBase 2.0 provides an additional opportunity to conduct concordant and differential pathway analysis for interpreting EV mRNA expression data and gaining insights into biological mechanisms. Third, the predicted abundances of EV origins will provide researchers with cellular-level indicators to evaluate the homogeneity and heterogenicity of different cohorts in physiological or pathological conditions. A few issues however should be noted: (i) The expression levels of most exLRs are comparatively low given the low abundance of EVs in human biofluids. Therefore, the expression frequencies are provided and act as additional parameters for expression evaluation. (ii) Compared with blood samples, the sizes of urine, CSF and bile samples are too small. In the future, more attention will be given to obtain exLR-seq data for such biofluids. (iii) The exLR-seq data of ESCC, GBM and MEL blood samples are produced by different teams with low mapped read counts. Hence, these data are only used to annotate exLRs detected in such types of samples, and the differences between the three groups and the healthy group were not taken into consideration. The sample selection criteria will be further refined to reduce the influence of sequencing quality on downstream analysis.

To our knowledge, exoRBase 2.0 remains the only online resource available for exploring different long RNA biotypes of EVs in human normal and cancerous biofluids. Based on these new features, we hope exoRBase 2.0 will be one of the most popular tools to facilitate the identification of novel exLR signatures from human biofluids and to help discover new circulating biomarkers for the improvement of disease diagnosis and therapy. The platform will be updated regularly in the future. We are considering adding exLR data of other biofluids, such as ascites, gastric fluid and saliva. We also encourage researchers to share exLR-seq data of human biofluids in the data submission section to enrich this repository and highlight their discoveries.
